# Rationale and Design of the MIA Trial: A Proof‐of‐Concept Study of Minimally Invasive Autopsy in an Infectious Cause of Death in a Scandinavian ICU Setting

**DOI:** 10.1111/apm.70190

**Published:** 2026-03-30

**Authors:** Fridtjof Gjerdrum Helgesen, Birgitte Grønkær Toft, Camilla Musaeus Donato, Ulf Gøttrup Pedersen, Thomas Hildebrandt, Xiaohui Chen Nielsen, Eric Santoni‐Rugiu, Gro Linno Willemoe, Lothar Wiese, Henning Bay Nielsen

**Affiliations:** ^1^ Department of Infectious Diseases Zealand University Hospital Denmark; ^2^ Department of Anesthesia and Intensive Care Zealand University Hospital Denmark; ^3^ Department of Pathology Copenhagen University Hospital ‐ Rigshospitalet Copenhagen Denmark; ^4^ Department of Clinical Medicine University of Copenhagen Copenhagen Denmark; ^5^ Department of Clinical Microbiology Zealand University Hospital Denmark

**Keywords:** Autopsy, MIA, Minimally, MITS, Post‐mortem

## Abstract

It remains a challenge to obtain well‐preserved tissue samples from deceased patients as access to regular autopsy is limited. Minimally invasive autopsy (MIA) is a potential alternative to the complete diagnostic autopsy because of its efficacy in providing non‐autolyzed tissue samples and its increased acceptability amongst the bereaved. The study follows an exploratory, prospective design without any kind of intervention. Inclusion criteria are deceased adults (≥ 18 years), confirmed signs of death, uncertain or multifactorial cause of death, present or previous infection, and permission from next of kin to perform MIA, defined by ultrasound (US)‐guided samples from the heart, lungs, liver, spleen, and kidneys using a TruCut semi‐automatic coaxial needle (14G; 16 cm length). Tissue samples, fixed in BiopSafe 20 mL formalin test tubes, are sent for pathological evaluation. The primary outcome is the achievement of well‐preserved tissue samples, representative of the target organ and suitable for histopathological evaluation and diagnosis. An exploratory outcome is to establish the degree of clinico‐pathological discrepancies between pre‐mortem clinical status and post‐mortem pathological descriptions. This investigator‐initiated study is designed to validate the feasibility of MIA to enable fast‐track post‐mortem evaluation in an ICU environment.

## 1 Background

Infectious diseases remain one of the leading causes of death worldwide, with WHO citing lower respiratory infections as the 8th highest cause of death (CoD) and COVID‐19 as the 2nd highest CoD in high‐income countries as of 2021 [1]. The complete diagnostic autopsy (CDA) is considered the gold standard for post‐mortem diagnosis as it provides quality control, essential feedback for clinicians, and highlights pathogenesis and epidemiology [2, 3, 4, 5, 6]. However, CDA procedures have steadily declined in recent decades. In Denmark, for instance, in the 1990s, the total autopsy rate was between 12% and 16%, but has since fallen to 4% [7]. In the UK, hospital autopsies only account for 1.2% of total autopsies since most autopsies are performed outside the hospital with a coroner's requisition [8].

Contributing factors to the decrease in CDA are an increased belief that the CoD is already known, cultural concerns surrounding disfigurement, and increased difficulties in obtaining consent from the bereaved. Other factors are time constraints, general procedure cost, and decreasing numbers of pathologists available to perform autopsies [4, 5, 9, 10, 11, 12, 13, 14]. However, despite improvements in technology, the frequency of missed disorders has stayed the same since the 1960s. This is seen especially in infectious diseases, particularly in early deceased septic ICU patients [3, 15, 16, 17].

Minimally invasive autopsy (MIA) is suggested as a feasible alternative to CDA because of lowered risk of infection in personnel [4, 5, 6, 10, 14, 18, 19, 20, 21]. In addition, it seems easier to obtain consent amongst the bereaved and as the post‐mortem interval is shortened, tissue autolysis is minimized [4, 5, 11, 14, 18, 22, 23, 24, 25]. Furthermore, the reduced cost of MIA compared to CDA coupled with an increased acceptability at the family and community level, has made the MIA approach popular in low‐income countries [4, 5, 6, 18, 23, 24, 26, 27]. Although the CHAMPS platform utilizes MIA for child mortality and stillbirth surveillance [28], it was recently reported that MIA, alongside TaqMan Array Cards (TACs), is useful in detecting pneumonia and sepsis in neonates in Southeastern Africa [29]. Moreover, up to 83% concordance between MIA and CDA in stillbirths and neonates was reported in Asian and African countries, supporting the usefulness of MIA [30, 31]. In adults, others suggested that MIA is suitable for detecting pneumonia and sepsis as aCoD [21].

During the COVID‐19 pandemic in high‐income countries, MIA gained popularity as an alternative to CDA since autopsies came to a halt because of safety concerns. During the pandemic, smaller case series supported MIA as a feasible technique to determine pathological changes to clarify the CoD. However, to our knowledge, the level of certainty in adults is sparse, and therefore more evidence seems appropriate. Secondly, there is a need for further studies to assess the practical biopsy procedure and the diagnostic performance of MIA before a routine set‐up is considered. If proven feasible, MIA could represent a scalable alternative to conventional autopsy in high‐income countries.

## 2 Methods

### 2.1 Study Design, Setting, Population, and Intervention

An exploratory, prospective study design without therapeutic intervention of any kind was developed. The study will be initiated at Zealand University Hospital, with an expected enrollment from November 2025 with a two‐year inclusion period. We plan to include deceased adults (≥ 18 years) with confirmed signs of death, uncertain or multifactorial cause of death, present or previous infection, including septicemia/bacteremia, where permission by next of kin to perform the procedure is obtained.

On the 25th of April 2025, the project was approved by the Regional Scientific Ethical Committee (SJ‐1097). The project adheres to the principles of Good Clinical Practice (GCP) and complies with regulatory requirements and guidelines. Participants will be enrolled according to Danish regulations, which allow informed consent by next of kin. We follow the normal procedures for collecting informed consent and any modifications to these as approved by the Regional Scientific Ethical Committee. For patients with whom the clinical course indicates a fatal outcome, the next of kin will be contacted with a request to include his or her relative in the current study. Such a request will be made with the highest discretion and respect for the autonomy of relatives, but also regarding the potential wishes of the deceased person. Inclusion of a patient in the current study will then begin to take place. All consenting parties will be provided with written and oral information regarding the study. It is preferable that a participant is alive at the time informed consent is given, and a consideration period of a minimum of 24 h is offered to next of kin.

Post‐mortem biopsies are planned as soon as possible after permission is granted by next of kin. Early signs of death (no breath and no heart rhythm as determined by cardiopulmonary stethoscope) are concluded by the responsible clinician. The responsible clinician determines when late signs of death (rigor mortis and/or livor mortis) can be declared.

A feasibility test was conducted during the COVID‐19 pandemic in a Danish ICU with inclusion of two patients. The goal of the test was to determine the suitability of the MIA method for determining pathological changes in tissues of certain organs. The initial case series was approved by the Regional Scientific Ethical Committee (SJ850). The initial pilot study reported that MIA appeared feasible in a Danish ICU to provide post‐mortem biopsies under an infection‐controlled regime [24].

**FIGURE 1:**
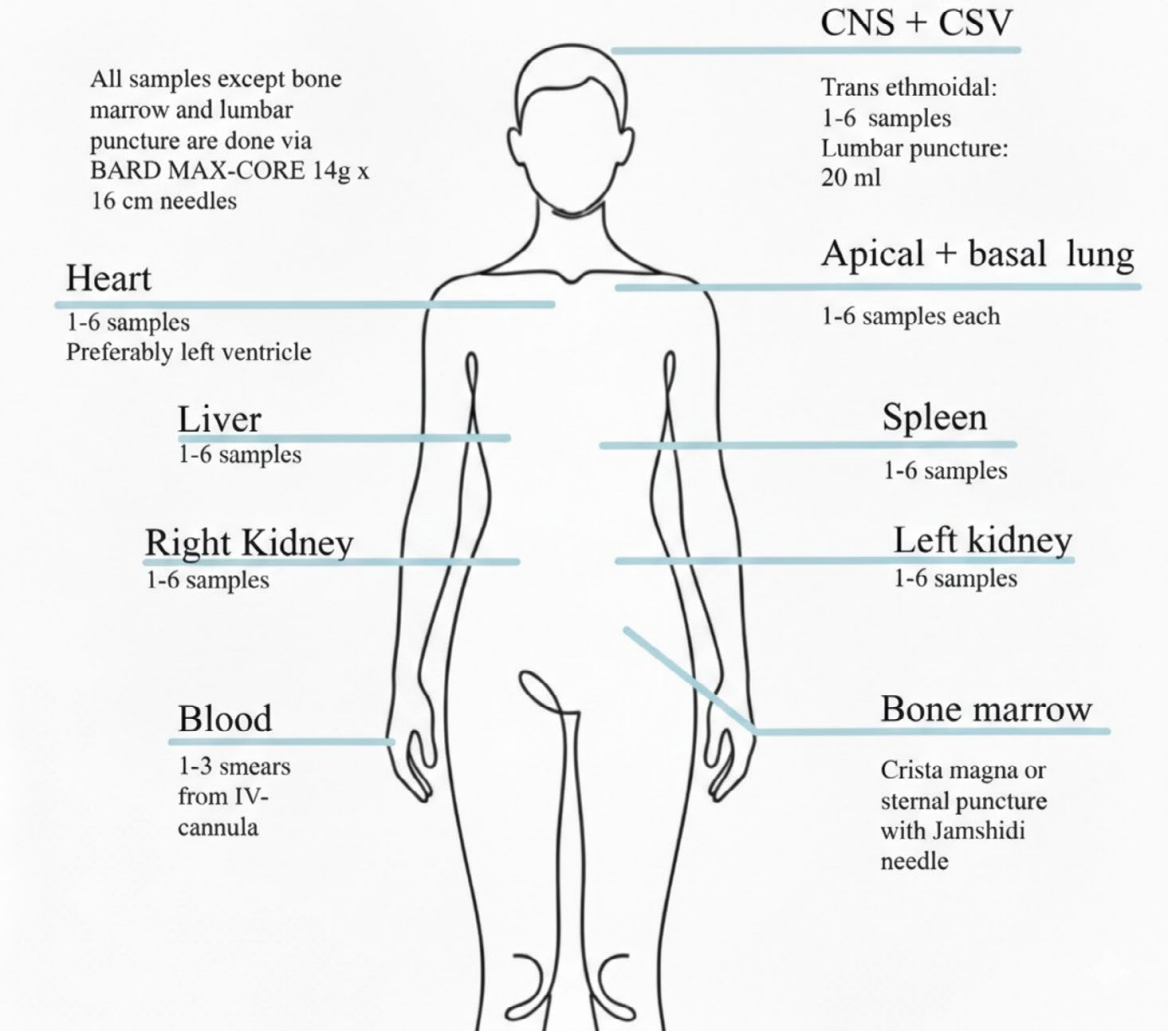
The figure illustrates the anatomical areas for targeted tissue sampling using MIA in deceased patients.

The MIA procedure applies US‐guided minimally invasive core biopsies (BARD MAX‐CORE, 14 G × 16 cm) from both kidneys (< 2 cm^3^, ≈0.15 mg), the lung (< 2 cm^3^, ≈0.15 mg), the liver (< 2 cm^3^, ≈0.15 mg), the heart (preferably myocardium from the left ventricle), and the spleen (< 2 cm^3^, ≈0.15 mg). Targeted biopsies of small tissue volume will be obtained, leaving the external integrity of the body almost intact, besides small lesions from biopsies that will be covered by dressing. Preferably, at least two biopsies from each organ will be obtained to ensure different tissue areas are represented for pathological examination. The obtained samples will immediately be placed in neutral buffered formalin using BiopSafe containers and stored at room temperature. Furthermore, the central nervous system will be sampled via trans‐ethmoidal puncture with the BARD MAX‐CORE, and bone marrow from the sternum will be sampled with a Jamshidi needle. Cerebrospinal fluid is taken with a spinal tap needle, and blood smears are taken from the IV cannula. Time of death, start of procedure, and end of procedure are noted to determine the post‐mortem interval and procedure time to evaluate the overall efficacy and feasibility of the procedure. Figure 1 illustrates the applied MIA procedure.

After fixation in formalin for at least 24 h and tissue processing, the samples will be paraffin‐embedded, and standard histochemical stains (hematoxylin–eosin stain, Alcian Blue‐Van Gieson stain for simultaneous visualization of acidic mucins/glycosaminoglycans and collagen/connective tissue) will be performed on slides with tissue sections to assess morphological changes by light microscopy. Tissue samples may be ad hoc assessed by additional specific histochemical and immunohistochemical staining and molecular analyses, such as fluorescence in situ hybridization and targeted next‐generation sequencing of gene panels. The pathological analysis is performed by specialists in Pathology at the Department of Pathology, Rigshospitalet, Denmark. The biopsy representativeness of the specific organ and quality is evaluated as the intended primary outcome for this trial.

Another set of samples for microbiological analysis will be taken following the same procedure. These samples are the first to be taken and will be stored at −80° in cryo‐safe containers. Clean biopsy needles will be used for each organ to avoid cross‐contamination. The samples will be used for microbiological evaluation, applying multiplex‐polymerase‐chain reaction (PCR) and/or microarray techniques.

Clinical data are registered prospectively in a RedCap database, where data related to infection, cardiovascular disease, and renal impairment, as well as need for respirator support and CoD as determined by affiliated clinicians, are added in a standardized manner. Consensus CoD will be determined via a panel of health care professionals (an anesthesiologist, an infectious disease specialist, a microbiologist, and a pathologist) on the basis of available data (clinical, microbiological, pathological), in accordance with WHO: The International Classification of Diseases, 11th revision (ICD‐11).

### 2.2 Rational, Aim, Hypothesis, and Outcome

It remains a challenge to obtain well‐preserved tissue samples from patients deceased during infectious disease as access to regular autopsy is limited. The aim of the MIA trial is to obtain representative, well‐preserved, high‐quality tissue samples from selected organs for histopathological examination and diagnosis in the context of infectious CoD.

The hypothesis of the study is that it is possible to organize a set‐up where post‐mortem biopsies can be obtained. Feasibility parameters include (i) obtaining informed consent and planning the procedure within 24 h after time of death, (ii) obtaining representative biopsies of the targeted organs, (iii) delivering well‐preserved (good quality) tissue samples to pathological examination (sample quality will be defined as good, less good, or bad), and (iv) establishing the degree of clinico‐pathological discrepancies between pre‐mortem clinical state and post‐mortem pathological descriptions.

An exploratory hypothesis is that the presence of multimorbidity (e.g., an interaction of diabetes, hypertension, obesity, and atherosclerosis) interacts with extensive organ support treatment (e.g., prolonged dialysis and mechanical ventilation) and is reflected in pathologic findings. The primary outcome is the achievement of well‐preserved tissue samples, representative of the target organ and suitable for histopathological evaluation and diagnosis. The secondary outcome measure is the correlation of the histopathological diagnosis to clinical status pre‐mortem. The exploratory endpoint is to evaluate whether the presence of premortem co‐morbidity disease, for example, obesity, diabetes, and hypertension, has consequences for post‐mortem pathological findings.

### 2.3 Statistics

The study is descriptive, although statistical evaluations may be applied when considered feasible. Here, relevant parameters are evaluated by *t*‐test for continuous variables and the chi‐square test for categorical variables. As the study is explorative in nature, a predefined sample size is difficult to determine. The initial pilot study included two patients; however, it failed to provide samples from several organs [24]. Especially the heart and the kidney appeared difficult to target. Thus, a cohort of 40 patients should be sufficient to provide sufficient and reliable data, considering the difficulties in obtaining tissue samples from all organs. This would allow us to make group comparisons between two patient characteristics (e.g., a comparison of morphology between patients with or without obesity, BMI ≥ 30). Using a sample size analysis on the basis of the occurrence of specific pathological findings of 20% in one group and app. 60% in the other group and applying the power of 0.80 and an alpha value of 0.05, it is justified to include 40 patients in this opportunistic, exploratory sample.

## 3 Discussion

This study aims to provide data regarding the practical biopsy procedure and the diagnostic performance of MIA under routine conditions in the context of an infectious CoD. If proven feasible, MIA could therefore represent a scalable and reliable alternative to the conventional autopsy technique in the ICU setting.

In ICU populations, mortality rates may be as high as 20%–30%, increased in septicemia; therefore, there is a need for a strengthened diagnostic process [32]. However, the discrepancy between premortem clinical diagnoses and post‐mortem findings is substantially higher in an ICU population compared with a hospital‐wide population [17]. In addition, septic ICU patients may have unresolved septic foci or even opportunistic infections that can be demonstrated at post‐mortem examination [3, 16]. Thus, in septic patients, an autopsy is an important diagnostic tool to identify CoD, which can deviate from clinical diagnoses, although clinical signs of myocardial infarction and pneumonia are usually difficult to ignore [15].

The importance of histopathological evaluations is underscored by the recent COVID‐19 pandemic. Here, autopsy studies revealed diffuse alveolar damage with hyaline membranes [22, 33] and the reports of pronounced venous thrombosis [34] prompted anticoagulant treatment for patients. Others documented tissue lesions related to co‐morbidity (hypertension, diabetes) and pronounced renal tubular damage [35]. A challenge is that the rate of regular autopsies is declining in Denmark. One cause is that fewer autopsies are requested by clinical physicians. In addition, relatives seem less willing to give their consent for a conventional gross autopsy [2, 12, 13]. A challenge with regular autopsy findings is that tissue samples are often obtained several hours or days after the patient has passed away. Rapid fixation of tissue in formalin is crucial to reduce post‐mortem tissue autolysis, which begins immediately after exchange of O_2_ and CO_2_ has stopped and compromises the recognition of diagnostic tissue changes. Moreover, the ability to detect viruses in tissue depends on the level of autolysis in samples [36].

Since MIA is performed by targeted needle biopsies, it is an attractive alternative to a regular autopsy [21]. Recently, a proof‐of‐concept study reported the organizational feasibility, efficiency, and safety of bedside post‐mortem minimally invasive tissue sampling in six ICU patients [4]. Many modern MIA protocols utilize a ‘blind’ sampling strategy, showing good performance, without the usage of US guidance and other imaging techniques such as CT or MRI [9, 14, 26, 37, 38]. However, we have chosen to utilize US since it is readily available in the ICU and most anesthesiologists are highly accustomed to its use from their training in US‐guided peripheral nerve blockade.

## 4 Conclusion

The conclusion is that the trial is considered to deliver information of relevance for the overall understanding of cases where infectious diseases are considered a likely CoD. Thus, post‐mortem examination results could provide clinicians with important learnings of disease development in ICU patients. It now needs to be evaluated in a larger cohort of patients before MIA can be considered a routine procedure in the clinical setting. An interesting perspective is that the presence of post‐mortem pulmonary and renal histopathological changes could assist in an evaluation of treatment guidelines in the ICU environment.

## Funding

The authors have nothing to report.

## Disclosure

*Timeline:* The first patient is expected to be included November 1st 2025 and the study database is expected to be closed November 31st 2027. The main manuscript will be submitted shortly thereafter.

## Consent

The authors have nothing to report. Trial results will be published in a peer‐reviewed scientific journal. Authorship will be based on scientific contributions to study design, conduct, analysis, or manuscript preparation.

## Conflicts of Interest

The authors declare no conflicts of interest.

## Data Availability

Results, regardless of the outcome, will be made available for public access. Data are aimed at being published in internationally recognized peer‐reviewed Journals. Also, if possible, oral presentation/poster presentations at Conference Meetings will be pursued. The Vancouver Declaration and the rules of good publication practice will be followed. Co‐authorship depends on the individual work done to conduct the study. Final co‐authorship will be decided by the project steering committee (see below).
